# Identification of a Novel Regulatory Mechanism of Nutrient Transport Controlled by TORC1-Npr1-Amu1/Par32

**DOI:** 10.1371/journal.pgen.1005382

**Published:** 2015-07-14

**Authors:** Mélanie Boeckstaens, Ahmad Merhi, Elisa Llinares, Pascale Van Vooren, Jean-Yves Springael, René Wintjens, Anna Maria Marini

**Affiliations:** 1 Biology of Membrane Transport, IBMM, Université Libre de Bruxelles, Gosselies, Belgium; 2 IRIBHM, Université Libre de Bruxelles, Campus ERASME, Brussels, Belgium; 3 Laboratoire des Biopolymères et des nanomatériaux supramoléculaires, Université Libre de Bruxelles, Brussels, Belgium; University College Dublin, IRELAND

## Abstract

Fine-tuning the plasma-membrane permeability to essential nutrients is fundamental to cell growth optimization. Nutritional signals including nitrogen availability are integrated by the TORC1 complex which notably regulates arrestin-mediated endocytosis of amino-acid transporters. Ammonium is a ubiquitous compound playing key physiological roles in many, if not all, organisms. In yeast, it is a preferred nitrogen source transported by three Mep proteins which are orthologues of the mammalian Rhesus factors. By combining genetic, kinetic, biochemical and cell microscopy analyses, the current study reveals a novel mechanism enabling TORC1 to regulate the inherent activity of ammonium transport proteins, independently of arrestin-mediated endocytosis, identifying the still functional orphan Amu1/Par32 as a selective regulator intermediate. We show that, under poor nitrogen supply, the TORC1 effector kinase' Npr1' promotes phosphorylation of Amu1/Par32 which appears mainly cytosolic while ammonium transport proteins are active. Upon preferred nitrogen supplementation, like glutamine or ammonium addition, TORC1 upregulation enables Npr1 inhibition and Amu1/Par32 dephosphorylation. In these conditions, as in Npr1-lacking cells, hypophosphorylated Amu1/Par32 accumulates at the cell surface and mediates the inhibition of specific ammonium transport proteins. We show that the integrity of a conserved repeated motif of Amu1/Par32 is required for the interaction with these transport proteins. This study underscores the diversity of strategies enabling TORC1-Npr1 to selectively monitor cell permeability to nutrients by discriminating between transporters to be degraded or transiently inactivated and kept stable at the plasma membrane. This study further identifies the function of Amu1/Par32 in acute control of ammonium transport in response to variations in nitrogen availability.

## Author Summary

Cells have evolved a variety of mechanisms to control the permeability of the plasma membrane to face environmental perturbations. Transcriptional regulation, endocytosis, gating and activity control of channels and transporters enable global or specific responses to stressful conditions and focused variations in nutrient availability. Emerging data from the yeast model reveal that the conserved TORC1 pathway regulates arrestin-mediated endocytosis of amino-acid transporters. We provide genetic and biochemical evidence for a novel mechanism enabling TORC1 to regulate the inherent activity of transport proteins via the Amu1/Par32 regulator intermediate. This low complexity protein mediates inhibition of specific proteins dedicated to the transport of ammonium, a favored nitrogen source, underscoring that TORC1 selects transporters to be degraded or transiently inactivated and preserved at the cell surface according to the environmental situation. The here-revealed mechanism of transport inhibition by Amu/Par32 is reminiscent to the inhibition of prokaryotic ammonium transport proteins mediated by P_II_-type proteins, key nitrogen signal transducers widespread in bacteria and Archaea.

## Introduction

Proteins of the Mep-Amt-Rh superfamily including the human Rhesus factors mediate the transmembrane transport of ammonium from bacteria to mammals [1–4]. Ammonium, hereafter referring to the sum of NH_4_^+^ and NH_3_, is a key nitrogen source for microorganisms and plants whereas it is mainly documented for its role as a blood pH regulator and for the deleterious consequences it has on the central nervous system upon cytotoxic accumulation in mammals for instance [5–7]. Mep-Amt-Rh proteins adopt a trimeric fold, with a proposed conducting pore crossing each of the three monomers [8–13]. The latter are composed of 11 or 12 helices and are prolonged by a cytosolic C-terminal extension showing conserved peculiarities specific to each of the Mep-Amt and Rh subfamilies [14]. In *Escherichia coli*, the cytosolic extension of each subunit of the AmtB trimers participates to the interaction with a trimeric association of the P_II_-type protein GlnK, a negative regulator of the ammonium transport activity [15,16]. Yet, P_II_-type proteins, key transducers of the nitrogen signal, are only found in bacteria, Archaea and a few plant species [17]. Unravelling the mechanisms and the signalling pathways involved in the activity regulation of eukaryotic Mep-Amt-Rh members remains an open field. We recently showed that the activity of Mep2, one of the three Mep-Amt-Rh proteins enabling ammonium transport in the yeast *Saccharomyces cerevisiae*, is dynamically controlled by the conserved TORC1 (Target Of Rapamycin Complex) pathway [18,19]. Under poor nitrogen supply, TORC1 downregulation relieves inhibition of the Npr1 kinase which enables phosphorylation of an autoinhibitory domain in the C-terminus of Mep2, thereby favouring ammonium transport. In contrast, preferred nitrogen supplementation activates TORC1 and thus inhibits Npr1. In this case, Mep2 is no longer phosphorylated and plasma-membrane phosphatases Psr1 and Psr2 additionally mediate desphosphorylation of preaccumulated phosphorylated Mep2, enabling autoinhibition of ammonium transport activity [18]. The latter mechanism notably contrasted with the known role of TORC1 and of its effector kinase Npr1 in regulating arrestin–mediated endocytosis of transporters but supported previous studies indicating a multiplicity in the mechanisms of Npr1-mediated regulation of *Saccharomyces cerevisiae* and *Candida albicans* Mep proteins [20–25]. Seminal works by Grenson and collaborators led to the isolation of mutations suppressing specific defects of Npr1-lacking cells in either amino-acid uptake, including the *mut-2*, *mut-4* and *mut-5* mutations, or in ammonium uptake, like the *amu1* mutation [25,26]. Of note the *mut* loci, also known as *npi1*, *npi2* and *npi3*, were shown to respectively encode variants of the Rsp5 ubiquitin-ligase, the orthologue of mammalian Nedd4, of the Doa4 ubiquitin-hydrolase, and of Bro1, the orthologue of mammalian Alix/Aip-1 [27–31]. While the characterization of the *mut* suppressor mutations shed light on the mechanism of ubiquitin-mediated endocytosis of permeases and the role of the multivesicular-body pathway in their delivery to the lysosome/vacuole [27–29,32,33], the nature of Amu1 and of the underlying mechanism of ammonium transport control remain unsolved.

Here, we cloned *AMU1* by functional complementation identifying *YDL173w*/*PAR32*, a gene of unknown function. We show that the phosphorylation status of Amu1/Par32 is dynamically controlled by TORC1-Npr1 in response to the quality of the nitrogen supply. When the function of Npr1 is inhibited, desphosphorylated Amu1/Par32 accumulates at the cell surface and mediates inhibition of specific ammonium transport proteins. This study unravels a novel mode of plasma-membrane permeability tuning, governed by TORC1 and reminiscent of the GlnK-mediated regulation of prokaryotic ammonium transport proteins.

## Results

### Mep1 and Mep3 are stable at the plasma membrane in Npr1-lacking cells

All three Mep ammonium transport proteins are most produced and active when wild-type cells are grown in the presence of a non-preferred nitrogen source, the *MEP2* gene being strongly expressed compared to *MEP1* and *MEP3* [19]. In these conditions, all three Mep transport activities largely depend on the integrity of the Npr1 kinase [22].

**Fig 1:**
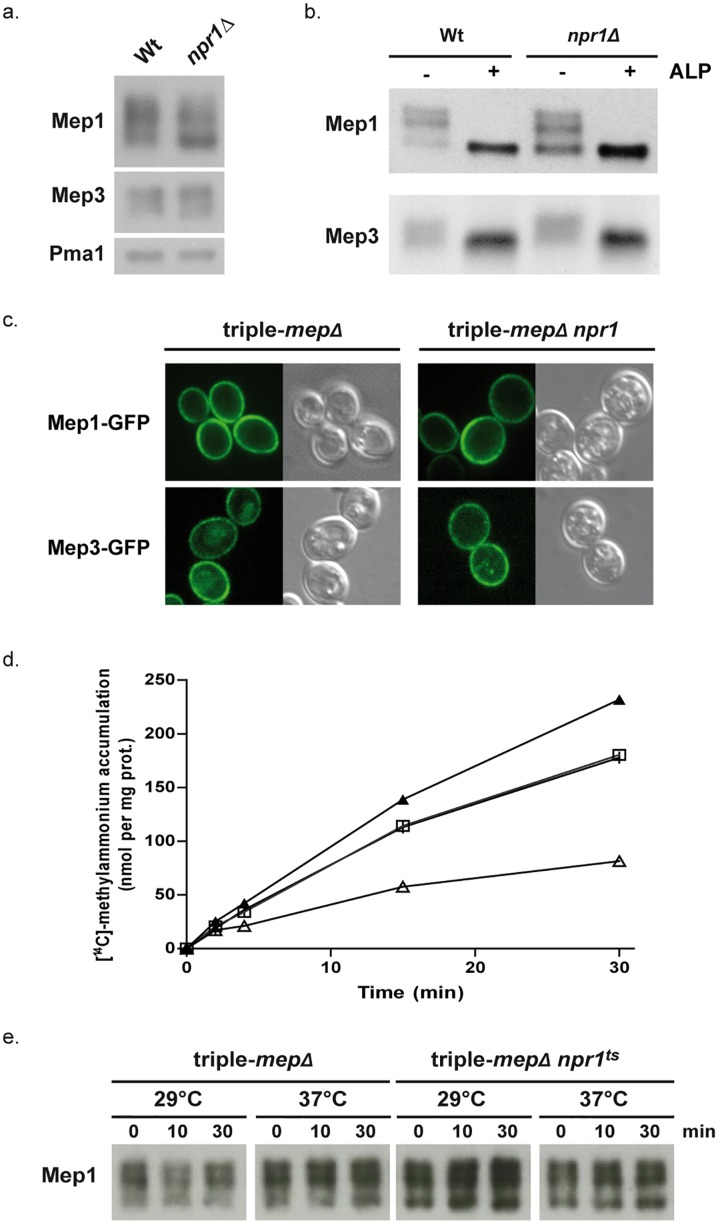
Npr1 is required for Mep1 and Mep3 inherent transport activity. (a) Immunodetection of Mep1 and Mep3 from membrane-enriched cell extracts. Wild-type (23344c) and *npr1Δ* (30788a) cells were grown in the presence of proline (0.1%) as nitrogen source. The plasma membrane proton ATPase Pma1 was detected as a loading control. (b) Immunodetection of Mep1 and Mep3 from membrane-enriched extracts treated (+) or not (-) with alkaline phosphatase (ALP). Wild-type (23344c) and *npr1Δ* (30788a) cells were grown in the presence of proline (0.1%) as nitrogen source. (c) Mep1-GFP and Mep3-GFP localization was observed by fluorescence microscopy in triple-*mepΔ* (31019b) and triple-*mepΔ npr1-1* (31052c) cells transformed with the pGAL1Mep1-GFP or pGAL1Mep3-GFP low-copy-number vectors and grown in the presence of proline (0.1%), galactose (3%) and glucose (0.3%). (d) [^14^C]-methylammonium (0.5 mM) accumulation in proline-grown triple-*mepΔ* (31019b,│,□) and triple-*mepΔ npr1* ^*ts*^ (MB063,▲,Δ) cells transformed with YCpMep1 after transfer in a similar medium preheated at 29°C (│,▲) or 37°C (□,Δ). (e) Immunodetection of Mep1 from membrane-enriched extracts of cells collected after the temperature shift as described in Fig 1d.

Npr1 is so far reported to protect amino-acid permeases from arrestin-mediated endocytosis and subsequent degradation, while we recently showed that the kinase also regulates the inherent activity of the Mep2 ammonium transport protein by controlling its phosphorylation state [18,20,21,34]. Upon addressing the influence of Npr1 on the protein levels of Mep1 and Mep3, we found that both proteins are not destabilized in the absence of the kinase (Fig 1a and 1b). Mep1 and Mep3 are respectively detected as at least 3 and 2 main forms, both in wild-type and Npr1-lacking cells. In the latter, the relative abundance of the 3 main Mep1 forms is slightly modified, the faster-running one being more predominant in the absence of the kinase. An essential requirement of Npr1 for the presence of one of these immunodetectable Mep1 and Mep3 forms can however not be deduced from these observations. We nevertheless tested whether the different detected Mep1 and Mep3 forms correspond to differentially phosphorylated states of the proteins. Treating protein extracts from wild-type and Npr1-lacking cells with alkaline phosphatase (ALP) led to a removal of the Mep1 and Mep3 slower-running forms, leaving one main intense state with a mobility similar to the faster-running form observed in the absence of ALP (Fig 1b). Hence, Mep1 and Mep3 exist under different phosphorylated states. Fluorescence microscopy revealed that Npr1 is dispensable for proper cell-surface localization of Mep1-GFP and Mep3-GFP as both proteins appeared evenly localized in the presence as in the absence of the kinase (Fig 1c).

Hence, Npr1 is required for Mep1 and Mep3 inherent transport activity but, in contrast to what has been previously shown for Mep2, the requirement of the kinase does not appear to be simply correlated to the detection of a particular phosphorylated status of the transport proteins.

### Npr1 inactivation correlates with Mep1 inactivation

We have previously shown that Npr1 integrity is notably required for the transport activity of Mep1 and Mep3 [22]. We used cells expressing a thermosensitive Npr1 variant [35] to test the consequence of immediate Npr1 inactivation on the Mep-dependent transport activity, focusing on Mep1. In contrast to the low affinity protein Mep3, the Mep1 transport activity can be easily monitored by following accumulation of [^14^C]-methylammonium (mea), a convenient tracer analogue of ammonium [19,22]. *MEP1* was expressed under the control of its own promoter from a low-copy-number centromeric plasmid (YCpMep1) in cells deprived of the three endogenous *MEP* genes (triple-*mepΔ*), bearing or not the thermosensitive Npr1 mutation (*npr1* ^*ts*^). Inactivation of the Npr1 kinase by shifting *npr1* ^*ts*^ cells at 37°C clearly reduced the Mep1-dependent accumulation of mea (Fig 1d). Moreover, the initial rate of mea uptake measured just after the shift showed an immediate effect of Npr1 inactivation on the Mep1 activity, revealing a rapid inactivation of Mep1 upon Npr1 inactivation (Triple-*mepΔ +* YCpMep1 at 29°C: 8.6 nmol min^-1^ per mg prot; triple-*mepΔ +* YCpMep1 at 37°C: 6.7 nmol min^-1^ per mg prot; triple-*mepΔ npr1* ^*ts*^ + YCpMep1 at 29°C: 8.4 nmol min^-1^ per mg prot; triple-*mepΔ npr1* ^*ts*^ +YCpMep1 at 37°C: 1.9 nmol min^-1^ per mg prot). Immunodetection of Mep1 did not reveal a major impact of Npr1 inactivation on the Mep1 protein level or bands distribution (Fig 1e).

These observations are consistent with Mep1 inherent activity being largely dependent on the maintenance of the Npr1 integrity, and indicate that the activity of plasma-membrane Mep1 is rapidly downregulated upon loss of Npr1 activity.

### Mep1 and Mep3 overproduction suppresses the Npr1 requirement

**Fig 2:**
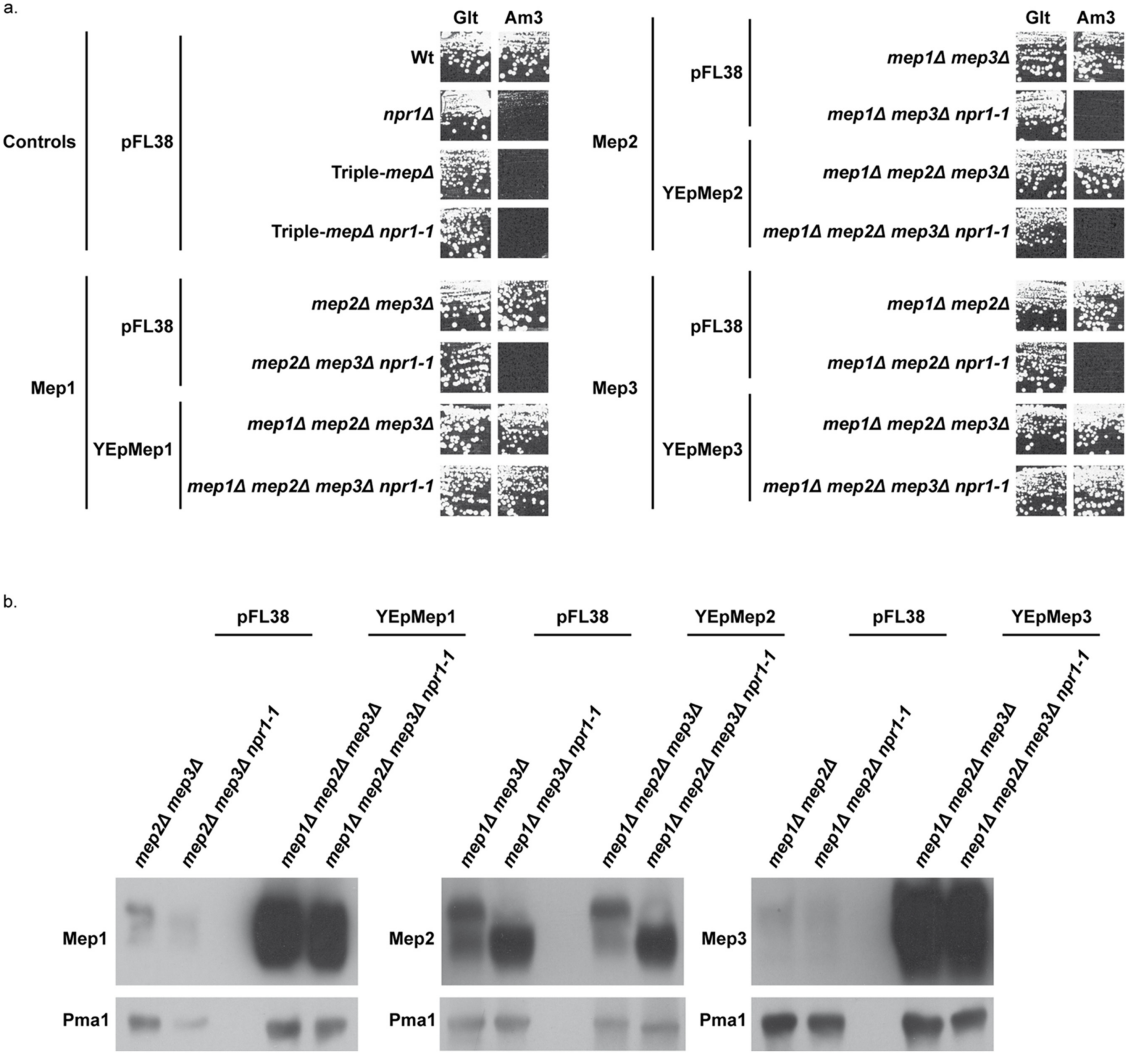
Mep1 and Mep3 overproduction suppresses Npr1 requirement. (a) Growth tests on solid medium containing, as the sole nitrogen source, ammonium 3 mM (Am3) or glutamate 0.1% (Glt, positive growth control). Wild-type (23344c), *npr1Δ* (30788a), *mep2Δ mep3Δ* (31018b), *mep2Δ mep3Δ npr1-1* (31059b), *mep1Δ mep3Δ* (31022a), *mep1Δ mep3Δ npr1-1* (31059d), *mep1Δ mep2Δ* (31021c) and *mep1Δ mep2Δ npr1-1* (31045c) cells were transformed with the empty vector pFL38. Triple-*mepΔ* (31019b) and triple-*mepΔ npr1-1* (31052c) cells were transformed with the empty vector pFL38 and with the high-copy-number plasmids: YEpMep1, YEpMep2 and YEpMep3. (b) Immunodetection of Mep1, Mep2 or Mep3 from membrane-enriched extracts. In the case of Mep2, the cellular extracts were treated with N-glycosidase F. Cells were grown in the presence of proline (0.1%) as nitrogen source. The plasma membrane proton ATPase Pma1 was detected as a loading control.

We next evaluated whether overproduction of each of the three Mep proteins could bypass the requirement of Npr1 for their transport function. The Mep functionality was assessed by performing growth tests in the presence of a low ammonium concentration as sole nitrogen source (Fig 2a). Triple-*mepΔ* cells are unable to grow in the presence of low ammonium concentrations while expression of only one of the three *MEP* genes enables growth in these conditions [19]. As expected, Mep1 and Mep3 proteins produced from the unique chromosomal *MEP1* or *MEP3* gene locus were fully dependent on Npr1 for function (Fig 2a) [22]. Expressing the *MEP1* and *MEP3* genes under their own promoter from a high-copy-number episomal plasmid led to an effective overproduction of the corresponding proteins (Fig 2b). In these conditions, growth on ammonium was observed despite the kinase absence (Fig 2a). *MEP2* expression from the episomal plasmid did however not allow Mep2 overproduction (Fig 2b), most likely reflecting the natural high strength of the *MEP2* promoter [19]. As expected in this case, the Mep2 function remained totally dependent on Npr1 (Fig 2a).

These data reveal that overexpression of the *MEP1* and *MEP3* genes relieves the Npr1 requirement for the corresponding Mep protein function.

### Identification of Amu1 as a negative regulator of ammonium transport

The above findings could be consistent with the existence of a limiting negative factor involved in the Npr1-mediated regulation of Mep1 and Mep3. In 1979, Dubois and Grenson characterized a strain bearing a suppressor mutation in a locus called *amu1* (ammonium uptake) enabling yeast growth on low ammonium concentrations despite the lack of a functional Npr1 [25]. Amu1 was proposed to act as a negative regulator of the low-affinity and high-capacity component of the ammonium transport activity of wild-type cells. These data could be consistent with Amu1 regulating Mep1 and/or Mep3, as both proteins possess a lower affinity for their substrate and a higher V_max_ compared to Mep2 [19].

**Fig 3:**
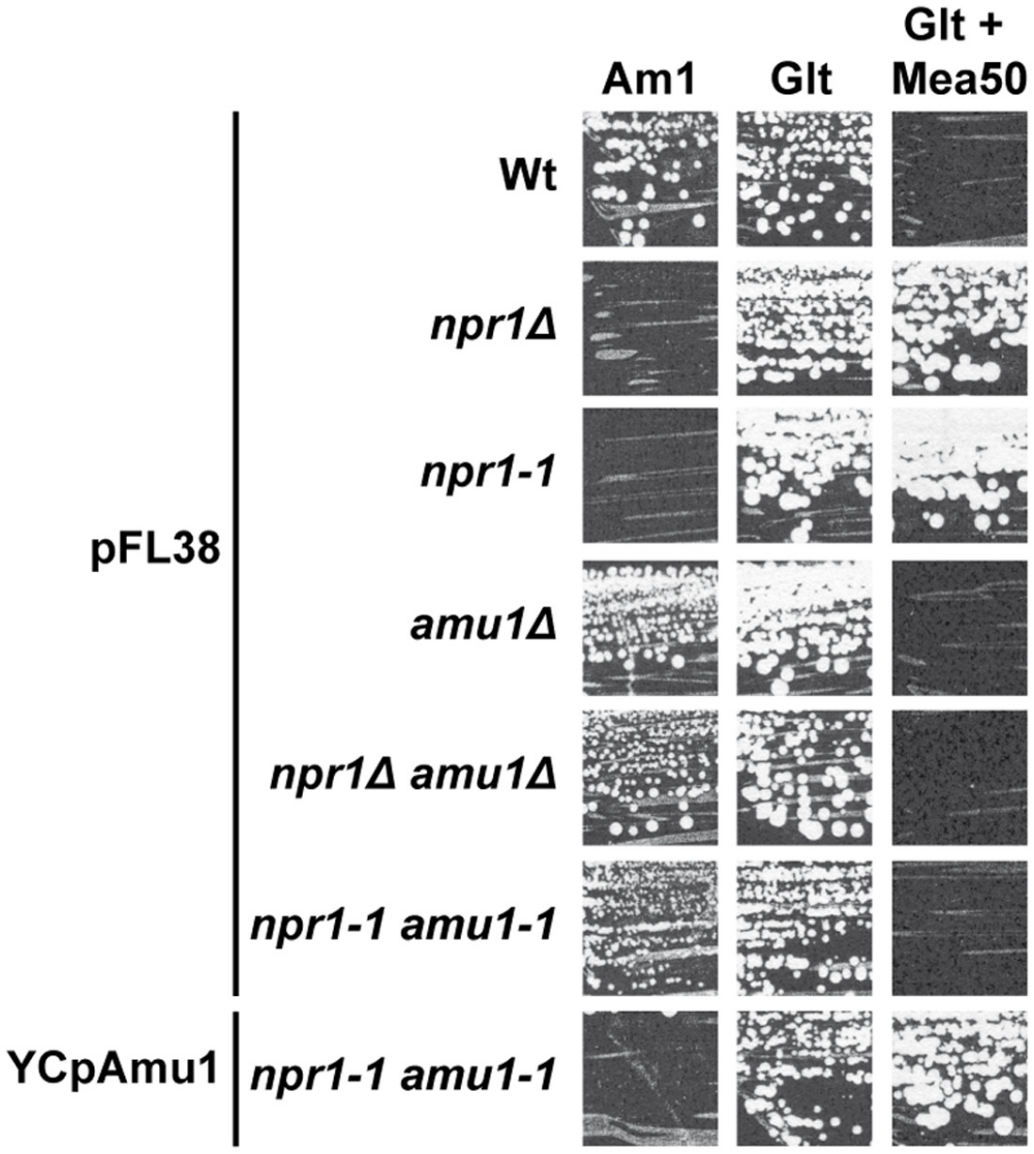
The *amu1* suppressor mutation restores growth of Npr1-lacking cells on ammonium. Growth tests on solid medium containing, as the sole nitrogen source, ammonium 1 mM (Am1) or glutamate 0.1% (Glt, positive growth control), supplemented or not with methylammonium 50 mM. Wild-type (23344c), *npr1Δ* (30788a), *npr1-1* (21994b), *amu1Δ* (MB139) and *amu1Δ npr1Δ* (36314b) cells were transformed with the empty vector pFL38. *amu1-1 npr1-1* (31034c) cells were transformed with the empty vector pFL38 and YCpAmu1.

Accordingly, while Npr1-lacking cells resist to toxic concentrations of methylammonium, a non-metabolizable ammonium analogue transported via Mep1 and Mep3, cells further bearing a mutated *amu1* locus do not grow in these conditions (Fig 3). We used the latter phenotype to clone the *AMU1* gene by functional complementation of *amu1-1 npr1-1 ura3* cells using a genomic library of the Σ1278b strain and selecting clones able to grow in the presence of a toxic concentration of methylammonium. The selected clones were next controlled for loss of growth on ammonium as sole nitrogen source (Fig 3). Analysis of the plasmidic content of these clones revealed that they all were transformed by a plasmid bearing one common gene, namely the *YDL173w* ORF.

Single expression of *YDL173w* from a centromeric plasmid complemented the *amu1-1* mutation in *amu1-1 npr1-1 ura3* cells, conferring resistance to methylammonium and loss of growth on low ammonium (Fig 3). Full deletion of *YDL173w* in Npr1-lacking cells was sufficient to restore growth on low ammonium and to confer sensitivity to methylammonium.

These findings are consistent with the *AMU1* gene corresponding to *YDL173w*, a gene coding for a protein of unknown function and previously named *PAR32* standing for ‘Phosphorylated After Rapamycin, 32kDa’ [36].

### The Amu1 protein contains a new repeated motif

**Fig 4:**
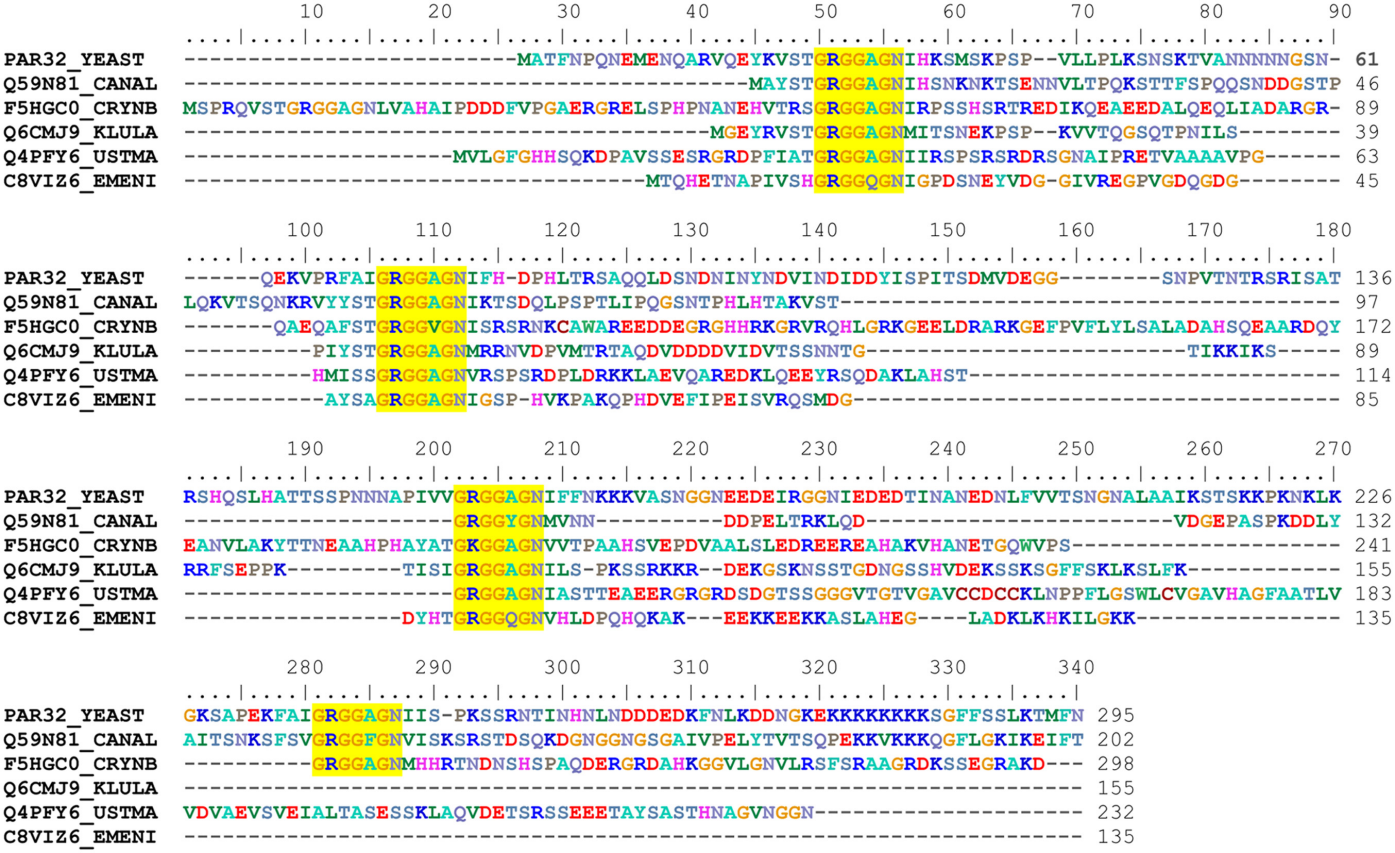
Amu1 is only conserved in fungi and contains a four-fold repeated motif. Sequences were retrieved from the UniProt database [38] primarily by a BlastP search [39] and secondly by a FuzzPro search [40] using the following searching pattern, G-R-G-G-X-[AG]-N-X(30,110)-G-R-G-G-X-[AG]-N. A selection of Amu1/Par32 orthologues is displayed in the alignment; the aligned sequences being denoted by their accession numbers: PAR32_YEAST, *Saccharomyces cerevisiae*, Q59N81_CANAL, *Candida albicans*, F5HGC0_CRYNB, *Crytococcus neoformans*, Q6CMJ9_KLULA, *Kluyveromyces lactis*, Q4PFY6_USTMA, *Ustilago maydis* and C8VIZ6_EMENI, *Aspergillus nidulans*. The multiple sequence alignment was automatically generated by ClustalW [41] and manually adjusted using BioEdit [42]. The repeated motif characterizing the Amu1 protein family is highlighted in yellow.

The biological function of Amu1/Par32 is unknown. A recent proteomic study identified the cleavage of the N-terminal methionine of Amu1/Par32 and the N-acetylation of the subsequent alanine [37], giving a predicted 294-residue long protein with a calculated molecular weight of 31.75 kDa. The Amu1/Par32 sequence exhibits a biased amino-acid composition with several low-complexity regions. In particular, poly-Asn and poly-Lys stretches are located in the N- and C-terminal region, respectively (Fig 4). The presence of such low-complexity regions often characterizes intrinsically disordered or natively unfolded proteins. Accordingly, disorder prediction algorithms predict an intrinsically unstructured protein based solely on the amino-acid sequence of Amu1/Par32. Of note, the Amu1/Par32 sequence contains an internal repeat ‘GRGGAGN’ present in four copies (Fig 4). No biological function is so far reported to be associated to this particular motif. Sequence similarities are difficult to reveal by simple blast analysis when considering proteins containing large low complexity regions. While one motif was not sufficient in itself, a multi-copy simultaneous search allowed the identification of orthologues, underlying the repetition of the motif as a common feature in the Amu1/Par32 protein family (Fig 4). All identified sequences were found in fungi, with a number of motif repetitions varying between 3 and 6 according to the considered protein.

### Amu1 is required for the Mep1 and Mep3 activity-loss occurring in Npr1-lacking cells

**Fig 5:**
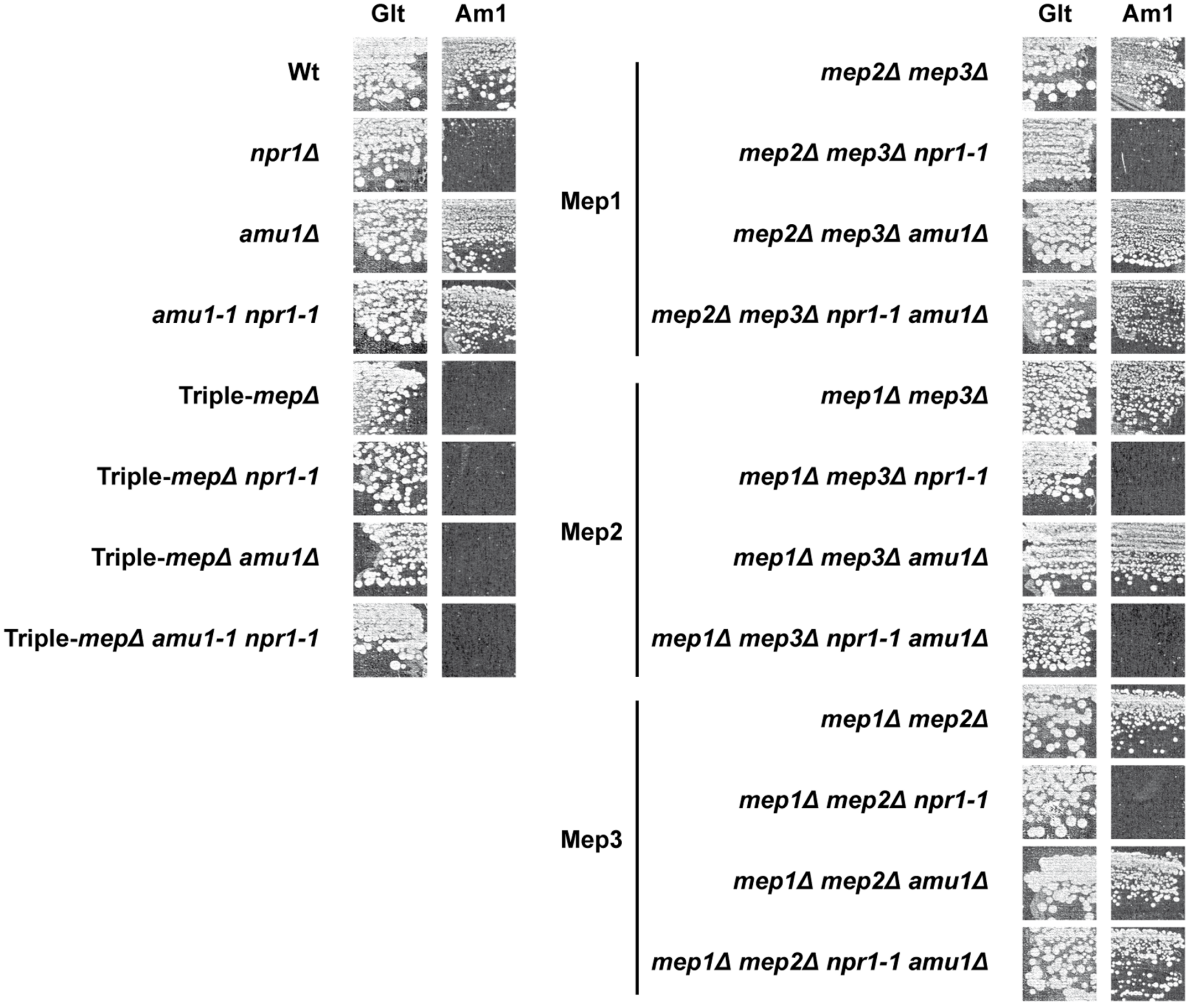
The *amu1* mutation restores the function of Mep1 and Mep3 in the absence of the Npr1 kinase. Growth tests on solid medium containing, as the sole nitrogen source, ammonium 1 mM (Am1) or glutamate 0.1% (Glt, positive growth control). Wild-type (23344c), *npr1Δ* (30788a), *amu1Δ* (MB139), *amu1-1 npr1-1* (31034c), triple-*mepΔ* (31019b), triple-*mepΔ npr1-1* (31052c), triple-*mepΔ amu1Δ* (AM114), triple-*mepΔ amu1Δ npr1-1* (31087c), *mep2Δ mep3Δ* (31018b), *mep2Δ mep3Δ npr1-1* (31059b), *mep2Δ mep3Δ amu1Δ* (PVV003), *mep2Δ mep3Δ amu1Δ npr1-1* (PVV001), *mep1Δ mep3Δ* (31022a), *mep1Δ mep3Δ npr1-1* (31059d), *mep1Δ mep3Δ amu1Δ* (PVV007), *mep1Δ mep3Δ amu1Δ npr1-1* (PVV005), *mep1Δ mep2Δ* (31021c), *mep1Δ mep2Δ npr1-1* (31045c), *mep1Δ mep2Δ amu1Δ* (PVV011) and *mep1Δ mep2Δ amu1Δ npr1-1* (PVV009) cells.

In order to determine which of the three Mep proteins recovers activity in a double *npr1 amu1* context, we constructed strains bearing the *AMU1* deletion, the *npr1* mutation and the deletions of two of the three *MEP* genes, in all the combinations. Deletion of *AMU1* improved growth on low ammonium of *npr1* cells expressing specifically *MEP1* or *MEP3*, but not those expressing the *MEP2* gene (Fig 5). The simple *AMU1* excision had no major impact on growth of cells producing one of the three Mep in the presence of a functional Npr1 kinase.

Measurements of initial uptake rates of [^14^C]-methylammonium were consistent with the growth tests (Table 1). An increase in Mep1 activity was observed in Npr1-lacking cells further deleted of *AMU1*, while Mep2 activity remained low in these conditions, as in *npr1* cells.

**Table 1:** Methylammonium uptake activity. Initial rates of [^14^C]-methylammonium (0.5 mM) uptake were measured. Wild-type (23344c), triple-*mepΔ* (31019b), *mep2Δ mep3Δ* (31018b), *mep2Δ mep3Δ npr1-1* (31059b), *mep2Δ mep3Δ amu1Δ* (PVV003), *mep2Δ mep3Δ amu1Δ npr1-1* (PVV001), *mep1Δ mep3Δ* (31022a), *mep1Δ mep3Δ npr1-1* (31059d), *mep1Δ mep3Δ amu1Δ* (PVV007), and *mep1Δ mep3Δ amu1Δ npr1-1* (PVV005) cells were grown in the presence of proline. The standard deviations are indicated for the values corresponding to the averages of two to six independent experiments.

Strains	[^14^C]-methylammonium uptake
	(nmol min^-1^ per mg prot.)
Wt	15.5 ± 1.9
*mep1Δ mep2Δ mep3Δ*	0.4 ± 0.3
***MEP1*** *mep2Δ mep3Δ*	11.9 ± 2.1
***MEP1*** *mep2Δ mep3Δ* ***amu1Δ***	10.2 ± 2.7
***MEP1*** *mep2Δ mep3Δ* ***npr1***	2.9 ± 0.7
***MEP1*** *mep2Δ mep3Δ* ***npr1 amu1Δ***	7.4 ± 1.8
*mep1Δ* ***MEP2*** *mep3Δ*	17.8 ± 4.3
*mep1Δ* ***MEP2*** *mep3Δ* ***amu1Δ***	14.4 ± 4.9
*mep1Δ* ***MEP2*** *mep3Δ* ***npr1***	1.1 ± 0.9
*mep1Δ* ***MEP2*** *mep3Δ* ***npr1 amu1Δ***	1.0 ± 0.8

Altogether, these observations reveal that loss of *AMU1* specifically restores the transport function of Mep1 and Mep3 in Npr1-lacking cells.

### Amu1 is phosphorylated in an Npr1-dependent manner

**Fig 6:**
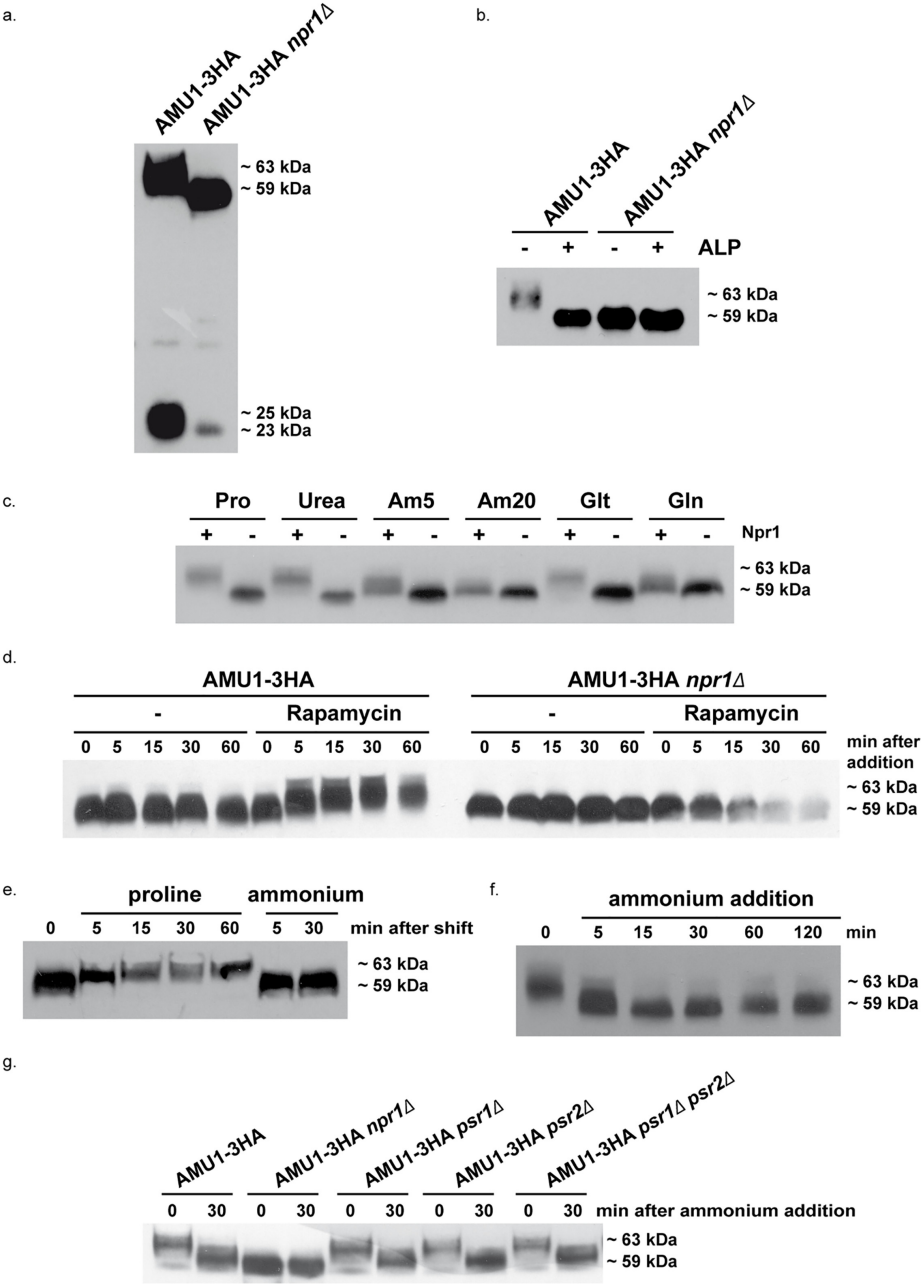
Amu1 phosphorylation is controlled by nitrogen supply, the Npr1 kinase and TORC1. Immunodetection of Amu1-3HA from total cellular extracts. (a-b) AMU1-3HA (MB142) and AMU1-3HA *npr1Δ* (36307b) cells were grown in the presence of proline (0.1%) as nitrogen source. (b) Total cell extracts were treated (+) or not (-) with alkaline phosphatase (ALP). (c) AMU1-3HA (MB142) and AMU1-3HA *npr1Δ* (36307b) cells were grown in the presence of proline (0.1%, Pro), urea (0.1%), ammonium (5 or 20 mM, Am), glutamate (0.1%, Glt) or glutamine (0.1%, Gln) as nitrogen sources. (d) AMU1-3HA (MB142) and AMU1-3HA *npr1Δ* (36307b) cells were grown in the presence of ammonium (20 mM) as nitrogen source. At time t = 0, rapamycin or the drug vehicle alone (-, control) was added to the cell culture. (e) AMU1-3HA (MB142) cells were grown in the presence of ammonium (20 mM) as nitrogen source. At time t = 0, cells were transferred in a proline-medium or in a similar ammonium-medium (control). (f) AMU1-3HA (MB142) cells were grown in the presence of proline (0.1%) as nitrogen source. At time t = 0, ammonium (20 mM) was added to the cell culture. (g) AMU1-3HA (MB142), AMU1-3HA *npr1Δ* (36307b), AMU1-3HA *psr1Δ* (PVV152), AMU1-3HA *psr2Δ* (PVV158), AMU1-3HA *psr1Δ psr2Δ* (PVV160) cells were grown in the presence of proline (0.1%) as nitrogen source. At time t = 0, ammonium (20 mM) was added to the cell culture.

We next tested whether the Npr1 kinase affects the phosphorylation state of the Amu1 protein. In proline-grown Npr1-containing cells, the Amu1-3HA protein produced from the chromosomal tagged-gene was detected as a principal signal of about 63 kDa (Fig 6a). In the latter experiment, overexposure of the autoradiogram also enabled to reveal a signal of about 25 kDa which was however not readily detectable in all the immunoblot experiments. In Npr1-lacking cells, these signals shifted respectively to about 59 and 23 kDa, the faster-running one being hardly detectable. The slower-running Amu1 form could correspond to a multimeric state of the protein or to a complex, both being resistant to denaturing conditions of the SDS-PAGE. Hereafter, when an immunoblot of Amu1-3HA will be discussed, we will mainly focus on the slower-running form.

ALP treatment of extracts from Npr1-containing cells was accompanied by a down-shift of the main Amu1-3HA signal from 63 to 59 kDa, thus at the same running-rate observed for the Amu1-3HA signal in Npr1-lacking cells extracts, treated or not with ALP (Fig 6b).

Hence, Amu1-3HA appears phosphorylated in an Npr1-dependent manner.

### TORC1 mediates tight nitrogen-dependent control of Amu1 phosphorylation

The Npr1 phosphorylation state and function is regulated by the TORC1 pathway according to the quality of the nitrogen supply [18,43–45]. Immunodetection of Amu1-3HA in Npr1-containing cells grown with nitrogen sources of different quality showed variations in the phosphorylation status of the protein (Fig 6c). The protein was the most phosphorylated during growth on poor nitrogen sources like proline and urea, or intermediate quality nitrogen source like glutamate, namely under conditions where Npr1 is hypophosphorylated and presumed active [44,45]. In contrast, Amu1-3HA appeared to be less phosphorylated during growth with raising concentrations of ammonium or with glutamine, conditions where Npr1 is hyperphosphorylated and inhibited in a TORC1-dependent manner. In Npr1-lacking cells, Amu1-3HA was immunodetected as a fast-running form whatever the nitrogen supply, indicating that the variation in phosphorylation state of Amu1-3HA is triggered by Npr1.

Rapamycin addition, to hinder TORC1 activity in cells growing on high ammonium concentration, was accompanied by a rapid up-shift of the Amu1-3HA signal, not observed in Npr1-lacking cells (Fig 6d). This is consistent with previous findings reporting that Amu1/Par32 is hyperphosphorylated after rapamycin treatment [46]. A similar rapid up-shift was also visible when ammonium-grown cells were transferred to a proline-medium, in keeping with a fast response of the Amu1 phosphorylation status to the quality of the nitrogen source (Fig 6e). Inversely, supplementation of a high ammonium concentration to proline-grown cells was correlated with a rapid down-shift of the Amu1-3HA signal consistent with instant dephosphorylation of Amu1 upon shift from non-preferred to preferred nitrogen supply (Fig 6f). We recently showed that the regulation of the inherent activity of Mep2 involves a dynamic control of the phosphorylation status of S457 in the C-terminal extension of the transport protein [18]. This control is mediated by a balance between Npr1-dependent phosphorylation and Psr1/Psr2-dependent dephosphorylation of Mep2. As the Mep1 and Mep3 negative regulation involves an intermediate protein which is phosphorylated in an Npr1-dependent manner, we tested whether Amu1 could be dephosphorylated via the Psr1 and Psr2 plasma-membrane phosphatases. Our data show that the Amu1-3HA ammonium-induced dephosphorylation was also observed in cells lacking one or both of the Psr phosphatases (Fig 6g). These findings further support a new distinction in the TORC1-Npr1 dependent mechanism of Mep2 regulation compared to Mep1/Mep3.

Altogether, these observations are consistent with TORC1 down-regulation triggering Npr1-dependent phosphorylation of Amu1 and with Amu1 phosphorylation state being tightly and rapidly regulated according to variation of the quality of the nitrogen supply.

### Amu1 localization is controlled by TORC1- and Npr1-dependent phosphorylation

**Fig 7:**
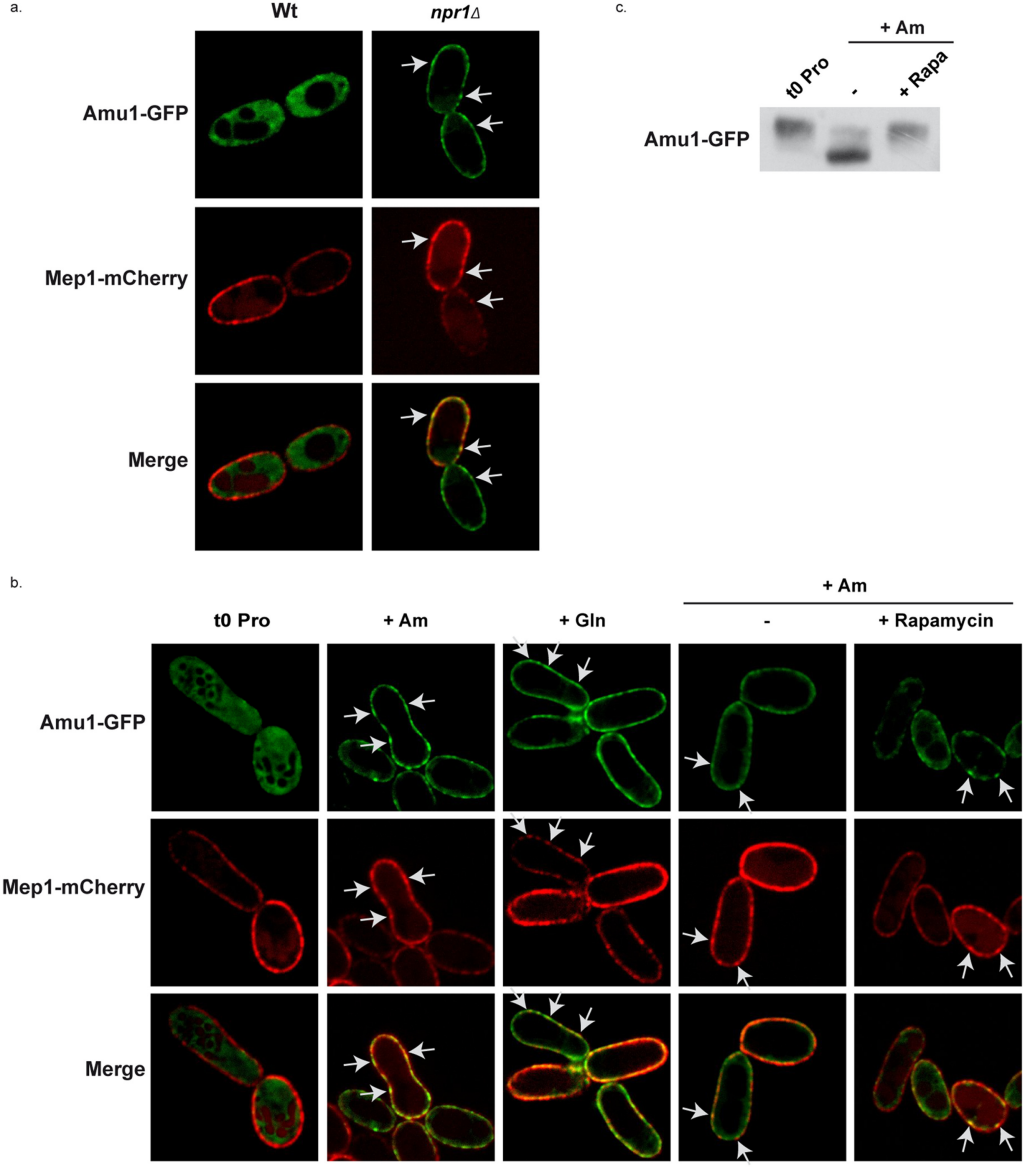
Amu1 localization is controlled by TORC1 and Npr1. (a-b) Amu1-GFP and Mep1-mCherry localizations were observed by fluorescence microscopy. *MEP1*-mCherry (MB321) or *MEP1*-mCherry *npr1Δ* (MB325) cells were transformed with YCpAmu1-GFP and grown in the presence of proline (0.1%). (b) *MEP1*-mCherry (MB321) cells transformed with YCpAmu1-GFP were grown in the presence of proline (0.1%) as nitrogen source. At time t = 0, ammonium (Am, 20mM) or glutamine (Gln, 0.1%) was added to the cell culture during 1h (2^nd^ and 3^rd^ columns). Rapamycin or the drug vehicle alone (control, -) was added to the proline-grown cells. 1h later, ammonium (Am, 20 mM) was added to the cell cultures during 1h (4^th^ and 5^th^ columns). (c) Immunodetection of Amu1-GFP from total cellular extracts of *amu1Δ* (MB139) cells transformed with YCpAmu1-GFP. Rapamycin or the drug vehicle alone (control, -) was added to proline-grown cells. 1h later, ammonium (Am, 20 mM) was added to the cell cultures during 1h.

It has been reported that an arrestin-target of Npr1 is specifically hypophosphorylated in Npr1-lacking cells where it accumulates at the plasma membrane [20]. We checked whether Amu1 could respond to the Npr1 regulation through a modified subcellular localization by analyzing the impact of the nitrogen supply and of Npr1 integrity on the localization of Amu1-GFP. In proline-grown cells, Amu1-GFP was most exclusively cytoplasmic in the presence of Npr1, whereas it was detected at the cell surface in Npr1-lacking cells (Fig 7a). Addition of either glutamine or high ammonium concentration to proline-grown Npr1-containing cells triggered an increase of Amu1-GFP at the plasma membrane and a decrease of cytosolic Amu1-GFP revealing a recruitment of the protein at the cell surface (Fig 7b). In all these conditions, Mep1-mCherry expressed from the tagged chromosomal *MEP1* locus was principally detected at the plasma membrane. Both Mep1-mCherry and Amu1-GFP labeled the cell surface with discontinuous fluorescence intensity, intense foci of Mep1-mCherry co-localizing with intense Amu1-GFP foci (Fig 7a and 7b). Addition of rapamycin to proline-grown cells prior ammonium supply at least partially prevented the recruitment of Amu1-GFP at the cell surface (Fig 7b), indicating that Amu1-GFP localization can be controlled by TORC1. Accordingly, the Amu1-GFP dephosphorylation induced by ammonium addition was inhibited in cells pretreated with rapamycin (Fig 7c).

### The localization of Amu1 depends on its phosphorylation state

Our data indicate that the kinase prevents plasma-membrane accumulation of Amu1, likely protecting Mep1 and Mep3 from the TORC1-dependent inactivation occurring upon preferred-nitrogen supplementation.

**Fig 8:**
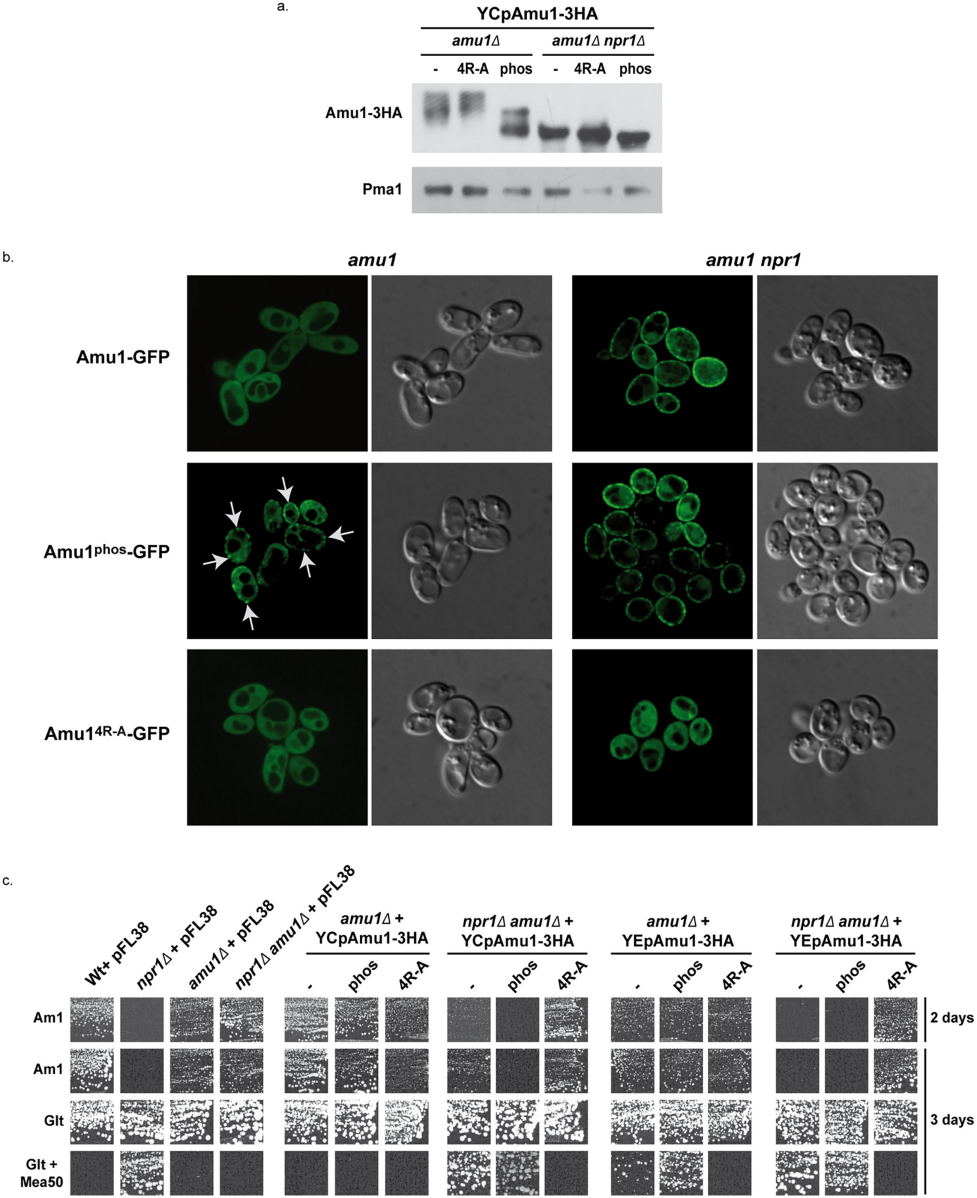
The phosphorylation state of Amu1 governs its localization and the integrity of the conserved motif is required for appropriate localization and inhibition function of the protein. (a) Immunodetection of Amu1-3HA from total extracts of proline (0,1%)-grown cells. *amu1Δ* (MB139) and *amu1Δ npr1Δ* (36314b) were transformed with YCpAmu1-3HA, YCpAmu1^4R-A^-3HA, and YCpAmu1^phos^-3HA. The plasma membrane proton ATPase Pma1 was detected as a loading control. (b) Amu1-GFP, Amu1^4R-A^-GFP and Amu1^phos^-GFP localization was observed by fluorescence microscopy. *amu1Δ* (MB139) or *amu1 npr1* (31034c or 36314b) cells were transformed with the corresponding YCpAmu1-GFP plasmid and grown in the presence of proline (0.1%). (c) Growth tests on solid medium containing, as the sole nitrogen source, ammonium 1 mM (Am1) or glutamate 0.1% (Glt, positive growth control), supplemented or not with methylammonium 50 mM (Mea50). Wild-type (23344c) and *npr1Δ* (30788a) were transformed with the empty vector pFL38. *amu1Δ* (MB139) and *amu1Δ npr1Δ* (36314b) cells were transformed with the empty vector pFL38, with the low-copy-number plasmids: YCpAmu1-HA, YCpAmu1^4R-A^-HA, YCpAmu1^phos^-HA, or with the high-copy-number plasmids: YEpAmu1-HA, YEpAmu1^4R-A^-HA, YEpAmu1^phos^-HA.

We next wished to address whether Amu1 dephosphorylation could constitute a signal for its cell-surface localization. The Amu1 295-residue-long protein contains no less than 29 serine, 15 threonine and 3 tyrosine residues and, at least 18 putative phosphorylation sites are predicted using the NetPhosYeast 1.0 server (http://www.cbs.dtu.dk/services/NetPhosYeast/). In addition, several independent large-scale phosphoproteomic analyses performed in response to different stimuli, such as rapamycin treatment, cell cycle regulation or general osmotic stress, have revealed numerous phosphorylated sites in Amu1, and also distinct combinations between these phosphorylated sites [36,46–51]. The Amu1 hyperphosphorylation observed upon rapamycin treatment or during growth on proline likely results from the simultaneous phosphorylation of several sites. Taking all these data into consideration, we decided to substitute by alanine 9 serine or threonine residues (S34, S36, S39, S49, S206, S246, S249, S250 and T253) including residues of sites reported to respond to rapamycin treatment [47]. The resulting Amu1-3HA variant, Amu1^phos^-3HA, was detected as a principal signal of about 59 kDa, showing a down-shift in the migration profile compared to the native Amu1-3HA protein (Fig 8a). The signal was composed of at least two major bands, the faster-running form being detected at a size similar to the Amu1-3HA signal observed in cells lacking the Npr1 kinase. These data indicate that the Npr1-dependent phosphorylation of Amu1^phos^-3HA is largely reduced.

While Amu1-GFP was mainly cytosolic in proline-grown cells, the Amu1-GFP variant mutated in the 9 potential phosphorylation sites, Amu1^phos^-GFP, was at least partially directed to the cell surface, despite the presence of the Npr1 kinase (Fig 8b), indicating that the reduction in Amu1 phosphorylation could be linked to its cell-surface localization.

We next addressed the functionality of the Amu1^phos^-3HA variant by performing growth tests on low ammonium medium as sole nitrogen source and on glutamate medium with a toxic methylammonium concentration (Fig 8c). Double *amu1Δ npr1Δ* cells expressing a functional Amu1 protein should behave as *npr1Δ* cells, thus showing no growth on ammonium and resistance to methylammonium, while expression of a non-functional Amu1 protein should not alter the growth of *amu1Δ npr1Δ* cells on ammonium and the sensitivity to methylammonium. The *AMU1-3HA* variants were expressed from centromeric (low-copy) and episomal (high-copy) plasmids. Expressing *AMU1-3HA* from a centromeric plasmid in *amu1Δ npr1Δ* cells partially inhibited growth on ammonium while it clearly conferred resistance to methylammonium, indicating that the Amu1-tagged protein is partially functional (Fig 8c). Overexpressing *AMU1-3HA* from an episomal plasmid in *amu1Δ npr1Δ* cells however fully complemented the *AMU1* deletion, suggesting that the partial function alteration of tagged Amu1-3HA can be compensated by overproduction of the protein. As expected, expression of *AMU1* ^*phos*^ *-3HA* in *amu1Δ npr1Δ* cells also complemented the *AMU1* deletion, indicating that the mutated protein has conserved its ability to inhibit ammonium transport (Fig 8c). *amu1Δ* cells producing or overproducing Amu1^phos^-3HA were able to grow on ammonium as the cells producing the non-mutated Amu1-3HA protein, showing that Amu1^phos^-3HA was still sensitive to Npr1-mediated inhibition. These data suggest that partial localization of Amu1^phos^-3HA at the cell surface in cells containing Npr1 is not sufficient to observe a constitutive inhibition of Mep1 and Mep3. However, *amu1Δ* cells overproducing Amu1^phos^-3HA appeared more resistant to methylammonium compared to those producing Amu1-3HA, suggesting that the hypophosphorylated variant may have gained a partial ability to inhibit Mep1 and/or Mep3 despite the presence of the Npr1 kinase. Expressing *AMU1* ^*phos*^ *-3HA* from a centromeric plasmid in *amu1Δ npr1Δ* cells appeared to induce a slightly stronger inhibition of growth on ammonium compared to *AMU1-3HA*, suggesting that the Amu1^phos^-3HA variant might have an enhanced inhibitory capacity on Mep1 and Mep3 even in the absence of the kinase.

Together, these results highlight the importance of Amu1 phosphorylation for the control of its localization, the hypophosphorylated Amu1^phos^-3HA variant being at least partially directed to the plasma membrane even if the Npr1 kinase is active. In these conditions, Amu1^phos^-3HA appears however unable to completely inhibit Mep1 and Mep3, indicating that it is still sensitive to Npr1-mediated inhibition, likely containing additional Npr1-dependent phosphorylation sites.

### Amu1 forms a complex with Mep1 and Mep3

Our data indicate that upon preferred nitrogen supply, Mep1 and Mep3 are inactivated in a TORC1- and Npr1-dependent manner via the Amu1 factor. As Amu1 is targeted to the plasma membrane in this condition, the protein could mediate inactivation of Mep1 and Mep3 via physical interactions.

**Fig 9:**
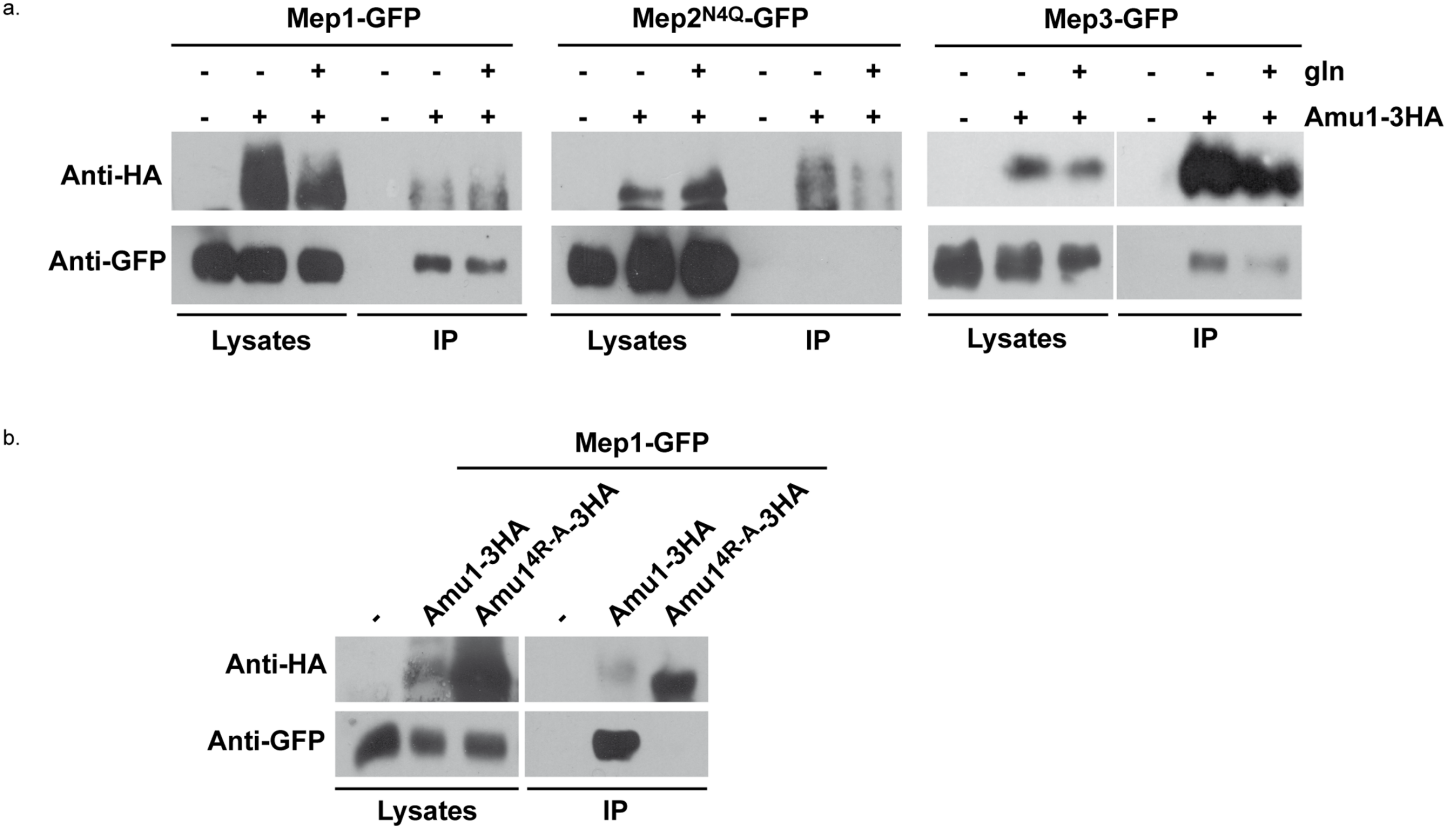
Amu1 interacts with Mep1 and Mep3 *in vitro*. After spheroplasting, the cells were lysed and Amu1-3HA was immunoprecipitated with anti-HA. Immunoblots of the lysates and immunoprecipitates (IP) with anti-HA and anti-GFP antibodies are shown. (a) Wt (23344c, negative control)) and AMU1-3HA (MB142) cells transformed with pGAL1Mep1-GFP, pGAL1Mep2^N4Q^-GFP or pGAL1Mep3-GFP were grown in the presence of proline (0.1%). Glutamine (gln, 0.1%) was added to AMU1-3HA (MB142) cells transformed with one of the 3 pGAL1Mep-GFP vectors (30 min). (b) Triple-*mepΔ* (31019b) cells transformed with pGAL1Mep1-GFP (negative control) and, triple-*mepΔ amu1Δ* (MB310) cells transformed with both pGAL1Mep1-GFP (LEU2) and YEpAmu1-HA (URA3) or with both pGAL1Mep1-GFP (LEU2) and YEpAmu1^4R-A^-HA (URA3) were grown in the presence of proline (0.1%).

We performed co-immunoprecipitation assays focusing on potential Amu1-Mep interactions. As Mep1 and Mep3 are poorly expressed proteins, we used an experimental setup enabling to produce Mep-GFP under the control of the strong *GAL1* promoter. We show that upon immunoprecipitation of Amu1-3HA, Mep1-GFP and Mep3-GFP were co-immunoprecipitated while Mep2-GFP was not (Fig 9a). It is to note that the co-immunoprecipitation of Mep1-GFP and Mep3-GFP with Amu1-3HA was observed when using proline-grown cells expressing Npr1 and that the addition of glutamine to the proline-grown cells had no significant impact on the efficiency of the co-immunoprecipitation, in the tested experimental conditions.

These results reveal that Mep1-GFP and Mep3-GFP are able to interact *in vitro* with Amu1-3HA.

### The integrity of the conserved and repeated motif of Amu1 is required for its function

We finally assessed the potential functional importance of the conserved repeated ‘GRGGAGN’ motif present in Amu1. We generated a plasmid allowing the expression of tagged Amu1-3HA where the arginine residue of all four motifs was mutated into alanine (Amu1^4R-A^-3HA). Amu1^4R-A^-3HA showed a migration profile similar to native Amu1, with no major alteration in the protein level of the major 63 kDa signal (Fig 8a). It also responded to Npr1 presence or absence similarly to non-mutated Amu1-3HA in terms of apparent phosphorylated and dephosphorylated states. However, Amu1^4R-A^-GFP was cytosolic both in the presence as in the absence of Npr1, revealing a key role of the motif in the cell-surface localization of Amu1 (Fig 8b). The functionality of the Amu1^4R-A^-3HA variant in the process of Mep1 and Mep3 inhibition was assessed by performing growth tests on low ammonium medium or on glutamate medium with a toxic methylammonium concentration (Fig 8c). Consistent with the mislocalization of Amu1^4R-A^-3HA, *amu1Δ npr1Δ* cells producing or overproducing Amu1^4R-A^-3HA remained able to grow on ammonium and sensitive to methylammonium, showing that this version of Amu1 was unable to inhibit Mep1 and Mep3. In addition, co-immunoprecipitation experiments further revealed that the *in vitro* interaction between Mep1 and Amu1 was compromised by the mutation of the four repeated motifs of Amu1 (Fig 9b).

These results show a crucial role for the arginine residue of the four repeated motifs in the cell surface localization of Amu1 and in the intrinsic ability of Amu1 to interact and to inhibit the Mep ammonium transport proteins.

Together, our findings are consistent with a direct negative role of Amu1 in the TORC1-Npr1 control of the inherent activity of Mep1 and Mep3.

## Discussion

**Fig 10:**
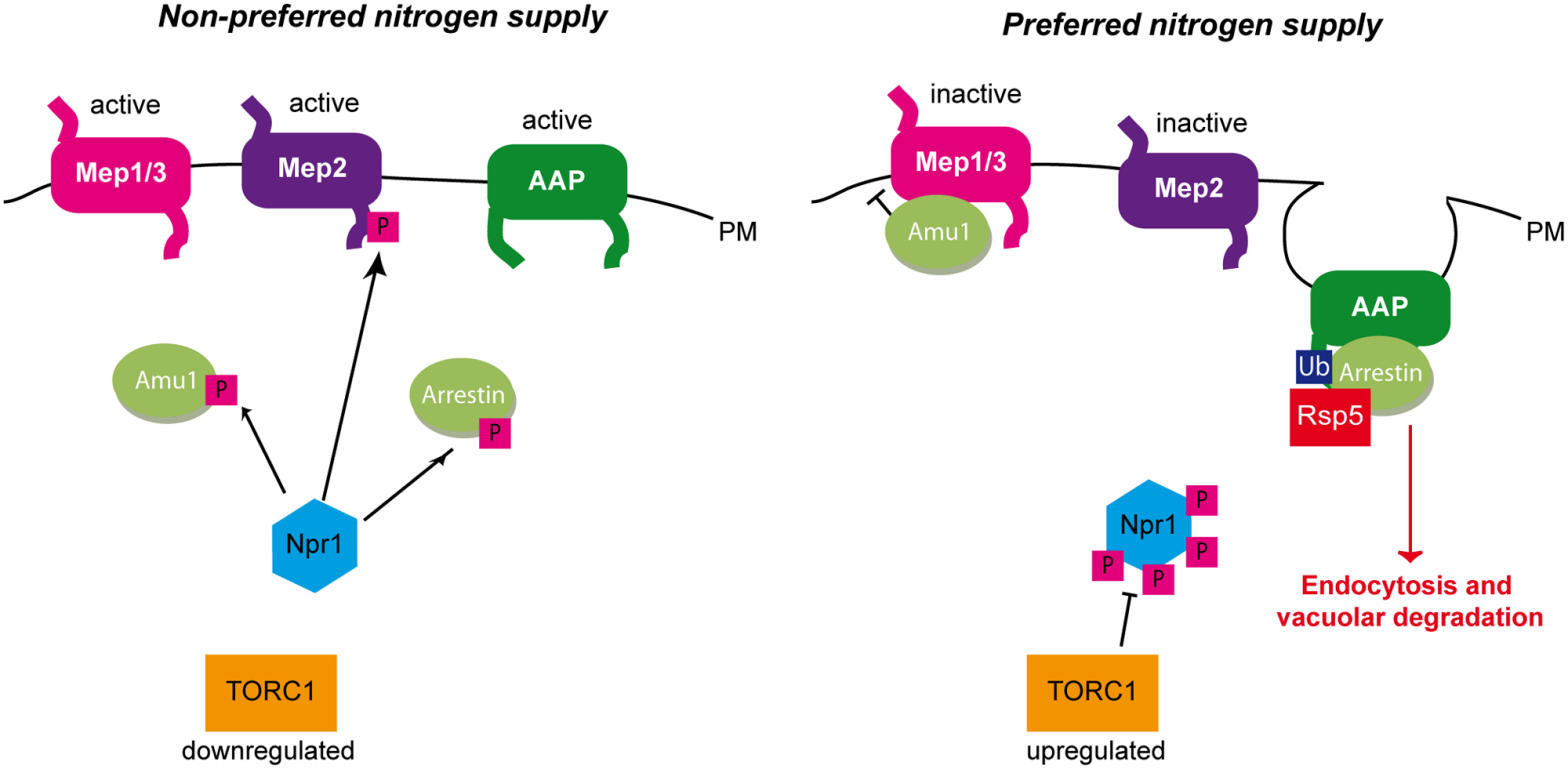
Three different strategies enable the TORC1-pathway to regulate plasma-membrane transport proteins. Under non-preferred nitrogen supply, TORC1 is poorly active and Npr1 is hypophosphorylated and able to mediate the phosphorylation of its different targets. Npr1 mediates the phosphorylation of Amu1 which remains cytosolic, while Mep1 and Mep3 are kept active. Npr1 enables C-terminal phosphorylation of the Mep2 ammonium transport protein, thereby silencing an autoinhibitory domain and allowing Mep2 activity. Npr1 mediates phosphorylation of arrestin-like adaptors thereby protecting their amino acid permease (AAP) targets from endocytosis and vacuolar degradation. Under preferred nitrogen supply, TORC1 is upregulated, Npr1 is hyperphosphorylated and inhibited. In these conditions, Amu1 is dephosphorylated, and accumulates at the cell surface, physically interacts with Mep1 and Mep3, and mediates inhibition of ammonium transport. The non-phosphorylated autoinhibitory domain of Mep2 prevents the enhancer domain to activate the transport protein. Dephosphorylated arrestin-like adaptors recruit the Rsp5 ubiquitin-ligase to their AAP targets, which are ubiquitylated, endocytosed and degraded in the vacuole.

This study reveals a novel mechanism enabling TORC1 and the effector kinase Npr1 to regulate nutrient permeability according to environmental variations. The TORC1-Npr1 pathway is thus able to regulate plasma-membrane transport proteins via three different ways (Fig 10). For instance, the TORC1-Npr1 pathway is so far mainly described for its control of the stability of amino-acid permeases at the plasma membrane [20,21]. Npr1 exerts a phospho-inhibitory control on arrestin-like adaptors thereby preventing recruitment of the Rsp5 ubiquitin-ligase to its permease targets, and consequently protecting them from endocytosis and vacuolar degradation. Seminal studies supported the existence of a different mechanism of Npr1-mediated regulation of yeast Mep ammonium transport proteins and also suggested the existence of a diversity among Npr1-dependent regulatory processes controlling Mep paralogues [22–25]. Consistently, we recently unraveled the molecular mechanism enabling TORC1-Npr1 to fine-tune the inherent activity of the Mep2 ammonium transport protein by dynamically regulating the phosphorylation status of an auto-inhibitory C-terminal domain of the transport protein [18]. Although Npr1 is also required for the activity of the two other Mep1 and Mep3 ammonium transport systems, our data reveal that their regulation indeed involves a different process implicating an inhibitory partner, Amu1/Par32, an ever still functional orphan. On the basis of our findings, we propose a model of Mep1 and Mep3 regulation by TORC1-Npr1 and the nitrogen supply (Fig 10). In the presence of a non-preferred nitrogen source, TORC1 is poorly active and Npr1 is hypophosphorylated and presumably active. In these conditions, Amu1 is phosphorylated in an Npr1-dependent manner and is mainly cytosolic while Mep1 and Mep3 are kept active. The Npr1 kinase might directly phosphorylate Amu1. For instance, a physical interaction between both proteins has been reported by two independent large-scale studies [52,53]. In the presence of a preferred nitrogen source, such as glutamine or a high ammonium concentration, TORC1 is upregulated, Npr1 is hyperphosphorylated and inhibited. In these conditions, Amu1 is dephosphorylated and accumulates at the cell surface. It forms a complex with Mep1 and with Mep3, and mediates inhibition of ammonium transport. Mep1 and Mep3 transport activity could be inhibited upon interaction with Amu1 by physical hindrance of the conducting pore crossing the hydrophobic core of the proteins. Alternatively, physical interaction with Amu1 might prevent C-terminal-dependent activation of transport. The C-terminal extension of several Mep-Amt proteins is indeed reported to regulate transport activation by controlling an allosteric switch [18,54,55]. It is also possible that Amu1 serves as a scaffold for yet to define regulatory protein(s), controlling the activity of Mep1 and Mep3.

A regulation by an inhibitory partner is highly reminiscent to the GlnK-mediated control of prokaryotic Mep-Amt proteins. GlnK belongs to the P_II_ protein family of signal transduction proteins widely distributed in bacteria and Archaea [17]. P_II_ proteins play a pivotal role in the control of nitrogen metabolism by regulating the activities of diverse enzymes, transcription factors and membrane transport proteins. Phylogenetic analyses indicate that P_II_ proteins evolved to regulate bacterial AmtB ammonium transport proteins, as evidenced by the early linkage of *GLNK* with *AMTB* in operon [56,57]. In *E*. *coli* cells, under nitrogen limitation, GlnK is uridylylated and essentially cytoplasmic, while supplementation of high ammonium concentrations is accompanied by deuridylylation of GlnK and interaction with AmtB at the plasma membrane [58]. Crystallisation of the AmtB-GlnK complex reveals that trimeric GlnK regulates trimeric AmtB by insertion of the regulatory deuridylylated T-loop of one GlnK monomers into the pore of the neighboring AmtB monomer in the trimer, and thus by physically blocking the substrate transport [15,16]. GlnK also binds α-ketoglutarate and ATP/ADP and, was recently shown to display an ATPase activity inhibited by α-ketoglutarate [15,16,59,60]. In conditions of nitrogen sufficiency, a drop in cellular α-ketoglutarate leads to a depletion of α-ketoglutarate bound to GlnK, accompanied by a hydrolysis of ATP into ADP. ATP hydrolysis would drive a change in the conformation of the T-loop allowing it to protrude deep into the cytoplasmic exit of the AmtB pore.

Amu1/Par32 is of unknown biochemical function. It is characterized by a four-fold repetition of a new motif ‘GRGGAGN’. The repetition of this motif enables to identify Amu1 orthologues, defining a new family of fungal proteins. Based on prediction algorithms, Amu1 would be classified as an intrinsically disordered protein. These algorithms do however not take into account possible post-translational modifications such as phosphorylation and the impact these could have on folding and function, as recently nicely demonstrated for another intrinsically disordered protein [61]. Although Amu1 is not related in sequence to P_II_ proteins, it is tempting to propose that it could acquire a particular folding/function upon binding to partners, such as the Npr1 kinase or the Mep proteins, and act as a functional analogue of the P_II_ proteins. For instance, we show that the hypophosphorylated Amu1^phos^ variant is at least partially directed to the plasma membrane even in the presence of Npr1. In contrast, the substitution by alanine of the conserved arginine residue in the four motifs in Amu1^4R-A^ prevents the cell-surface localization, normally occurring in the absence of Npr1, and this, despite Amu1^4R-A^ being dephosphorylated in these conditions. These data suggest that the integrity of the motifs plays a dominant role for the cell-surface localization of dephosphorylated Amu1. Moreover, if Amu1^4R-A^ has lost its ability to inhibit Mep1 and Mep3 *in vivo*, as expected given its mislocalization, Amu1^4R-A^ has also lost its proper capacity of interaction with Mep1 *in vitro*, indicating a key role of the conserved arginine residues. The motifs integrity could thus be required for Amu1 to adopt a particular folding enabling interaction with the Mep proteins. In this model, nutrient-induced dephosphorylation of Amu1 would favor an Amu1 folding process involving the repeated motifs. The dephosphorylation of Amu1 could also be required for the interaction with proteins involved in its cell-surface targeting.

We show that yeast cells have evolved different mechanisms to regulate transport proteins of the same family. While Mep2 shares about 40% of identity with Mep1 and Mep3, the two latter proteins share about 79% identity [19] and likely emerged from the whole-genome duplication event that occurred in the hemiascomycete branch [62]. We previously showed the existence of two functional Mep-Amt subfamilies in fungi distinguishable according to whether the first of two conserved histidines in the conducting pore is preserved, as in yeast Mep2, or replaced by glutamate, as in Mep1 and Mep3 [63]. In fungi, there is usually coexistence of at least one Mep2-type protein with a Mep1/3-type protein. In baker yeast, these two subfamilies notably differ in their kinetic properties and particularly in their optimal pH of transport. We previously proposed that Mep2 and Mep1/Mep3 could transport ammonium via different molecular mechanisms, involving or not a deprotonation step of the recognized NH_4_^+^ substrate, leading to opposite direct effects on intracellular pH and to different impacts on physiology. Of note, Mep2-type proteins have a unique status in that they are proposed to play a signalling role in filamentation induction, a dimorphic transition often associated to the virulence of pathogenic fungi [64]. These observations indicate a separation in the evolution history and a functional specialization of proteins of both subfamilies. Although both Mep2 and Mep1/3 regulations are mediated by TORC1-Npr1, evolution of different molecular mechanisms of activity control could enable to discriminate between both subfamily members and to fine-tune each regulatory process in response to specific physiological parameters. Interestingly, in *Candida albicans*, the CaMep2 activity is abolished in cells lacking CaNpr1 while the CaMep1 activity is only partially compromised, and *CaMEP3* appears to be a non-functional gene [24]. Though the potential role of the CaAmu1 protein (Fig 4) in CaMeps regulation is unknown, these data could point to variations to Npr1-dependency of Mep proteins among yeast species. However, it is also conceivable that the distinct functions carried out by Npr1 in *Saccharomyces cerevisiae* could be supported by distinct Npr1-like, or even distinct kinases, in other species. For instance, in collaboration with the team of Bettina Tudzynski, we recently showed that the expression of at least one of the *Fusarium fujikuroi* Npr1-like proteins confers growth on ammonium to the *Saccharomyces cerevisiae npr1* mutant while being unable to mediate Mep2 phosphorylation [65]. These data suggest that the *Fusarium* kinase is able to protect yeast Mep1 and/or Mep3 against Amu1-mediated inactivation while being unable to control Mep2 activity.

The TORC1-Npr1 control passing via the Amu1 intermediate affects at least two yeast transport proteins. The latter are dedicated to the transport of a preferred nitrogen source further underlying that TORC1-Npr1 discriminates between transporters to be degraded, transiently inactivated or kept active at the cell surface by controlling at least three different regulatory mechanisms. It will be informative to determine the specter of action of Amu1. Available data already indicate that Amu1 does not control amino-acid permeases like Gap1 and Put4 [25].

Could an Amu1-like regulatory mechanism also apply to mammalian ammonium transport proteins of the Mep/Amt/Rh family? To date the mechanisms and the pathways involved in the activity regulation of Rhesus factors, mammalian counterparts of yeast Mep proteins, are largely unknown. The human RhCG protein shares a similar structure with Mep/Amt and Rh proteins from bacteria and Archaea [8–13]. Recent characterization of a nsSNP variant of human RhCG indicated that the activity regulation of this Rhesus factor could implicate molecular mechanisms similar to those controlling their Mep-Amt homologues [14]. P_II_ proteins are absent from eukaryotes except their presence in plastids of a few plants, and obvious Amu1 homologues are limited to fungi. However, our findings suggest that the molecular mechanism of prokaryotic and fungal Mep-Amt proteins regulation is conserved although the regulatory partners are structurally different. It is tempting to propose that a similar mechanism has evolved to enable the regulation of mammalian Rhesus factors. Determining whether functional analogues of Amu1 and/or P_II_ exist in mammals constitute a challenge for further investigation.

## Materials and Methods

### Strains, growth conditions

The *S*. *cerevisiae* strains used in this study are listed in Table 2. All strains are isogenic with the wild type Σ1278b [66]. Cell transformation and gene deletions were performed as described previously [67,68]. mCherry-tagging of *MEP1* at the chromosomal locus was performed by homologous recombination using a PCR fragment amplified from the pFA6a-link-yomCherry-Kan plasmid, a gift from Wendell Lim and Kurt Thorn (Addgene plasmid 44903) [69]. HA-tagging of *AMU1* at the chromosomal locus was similarly performed using a PCR fragment amplified from pFA6a-3HA-kanMX6 [70]. Cells were grown in a minimal buffered (pH 6.1) medium with 3% glucose as the carbon source [71]. In experiments in which genes were expressed under the *GAL1* promoter, 3% galactose + 0.3% glucose were used for transcriptional induction, and 3% glucose for repression. To this medium, nitrogen sources were added as required by the experiment and as specified in the text. The nitrogen sources used were 0.1% proline, 0.1% urea, 0.1% glutamine, 0.1% glutamate, or (NH_4_)_2_SO_4_ at the specified concentration. When required, the medium was supplemented with 0.0025% uracil to complement the auxotrophy.

**Table 2:** List of strains used in this study.

Strain	Genotype	Source or reference
21994b	*npr1-1 ura3*	[72]
23344c	*ura3*	Lab collection
30788a	*npr1Δ ura3*	[34]
31018b	*mep2Δ mep3Δ ura3*	[19]
31019b	*mep1Δ mep2Δ*::*LEU2 mep3Δ*::*KanMX2 ura3*	[19]
31021c	*mep1Δ mep2Δ ura3*	[19]
31022a	*mep1Δ mep3Δ ura3*	[19]
31034c	*npr1-1 amu1-1 ura3*	[73]
31045c	*mep1Δ mep2Δ npr1-1 ura3*	[22]
31052c	*mep1Δ mep2Δ*::*LEU2 mep3Δ*::*KanMX2 npr1-1 ura3*	[22]
31059b	*mep2Δ mep3Δ npr1-1 ura3*	[22]
31059d	*mep1Δ mep3Δ npr1-1 ura3*	[22]
31087c	*mep1Δ mep2Δ mep3Δ npr1-1 amu1Δ ura3*	This study
36307b	*AMU1-3HA npr1Δ ura3*	This study
36314b	*amu1Δ npr1Δ ura3*	This study
AM114	*mep1Δ mep2Δ mep3Δ amu1Δ ura3*	This study
MB063	*mep1Δ mep2Δ mep3Δ npr1^ts^ ura3*	[18]
MB139	*amu1Δ ura3*	This study
MB142	*AMU1-3HA ura3*	This study
MB310	*mep1Δ mep2Δ mep3Δ amu1Δ leu2 ura3*	This study
MB321	*MEP1-*mCherry *ura3*	This study
MB325	*MEP1-*mCherry *npr1Δ ura3*	This study
PVV001	*mep2Δ mep3Δ amu1Δ npr1-1 ura3*	This study
PVV003	*mep2Δ mep3Δ amu1Δ ura3*	This study
PVV005	*mep1Δ mep3Δ amu1Δ npr1-1 ura3*	This study
PVV007	*mep1Δ mep3Δ amu1Δ ura3*	This study
PVV009	*mep1Δ mep2Δ amu1Δ npr1-1 ura3*	This study
PVV011	*mep1Δ mep2Δ amu1Δ ura3*	This study
PVV152	*psr1Δ AMU1-3HA ura3*	This study
PVV158	*psr2Δ AMU1-3HA ura3*	This study
PVV160	*psr1Δ psr2Δ AMU1-3HA ura3*	This study

Rapamycin (LC Laboratories) was used at a final concentration of 2 μg/ml from a stock solution prepared in 90% ethanol/10% Tween-20.

### Cloning of AMU1

The *S*. *cerevisiae AMU1* gene was cloned by screening a low copy number library [1], representing the genome of Σ1278b strain, for plasmids complementing the *amu1-1* mutation in the 31034c recipient strain (*npr1-1 amu1-1 ura3*). Cells transformed with the plasmid library were plated on a selection minimal medium containing glutamate 0.1% as nitrogen source and 50 mM methylammonium. Among about 125.000 transformants, 10 tested candidates had simultaneously recovered methylammonium resistance and loss of growth on low ammonium (1mM). DNA isolated from these transformants was transformed in *E*. *coli* JM109 for purification and amplification. The phenotypes were verified after reintroducing the 10 purified plasmids into 31034c cells. Sequencing of the 10 genomic inserts enabled to identify one single common gene, *YDL173w*. A HincII-HindIII DNA fragment, containing the single *YDL173w* ORF with 311 pb upstream and 121 pb downstream sequences, was subcloned into pFL38 [74] to generate YCpAmu1. The latter plasmid was sufficient to complement the *amu1-1* mutation of 31034c cells.

### Plasmids

Plasmids used in this study are listed in Table 3. Primers used in this study are available upon request. pGAL1Mep1-GFP: the *MEP1* gene was amplified by PCR using the YCpMep1 vector [1] as template and then cloned by *in vivo* recombination in the pGAL1Gap1-GFP vector [34] by replacement of the *GAP1* gene. pGAL1Mep3-GFP: the *MEP3* gene was amplified by PCR using YCpMep3 vector [19] as template and then similarly used to replace the *GAP1* gene in the pGAL1Gap1-GFP vector [34]. YEpMep1, YEpMep2 and YEpMep3 were constructed by transferring the complete insert of YCpMep1 [1], YCpMep2 [19] and YCpMep3 vectors [19] into the pFL44 vector [74] by restriction-ligation. YCpAmu1-GFP: a fragment containing the *AMU1* gene and promoter was amplified by PCR using *S*. *cerevisiae* (23344c) genomic DNA and then cloned by *in vivo* recombination in the YCpMep2-GFP vector [18] by replacement of the *MEP2* gene and promoter. YCp and YEpAmu1-3HA: a fragment containing a part of *AMU1-3HA* gene and terminator was amplified by PCR using MB142 (*AMU1-3HA*) genomic DNA and then cloned by *in vivo* recombination in the YCp or YEpAmu1 vector. Mutated *AMU1* ^*4R-A*^ and *AMU1* ^*phos*^ genes were synthesized and cloned in pUC57 vector by GeneCust (Dudelange, Luxembourg). YCp/YEpAmu1^4R-A^-3HA and YCp/YEpAmu1^phos^-3HA were constructed by transferring the complete insert of the corresponding pUC57-Amu1 into the YCp/YEpAmu1-3HA vector by restriction-ligation. YCpAmu1^4R-A^-GFP and YCpAmu1^phos^-GFP: a fragment containing a last part of *AMU1* gene fused to GFP was amplified by PCR using YCpAmu1-GFP and then cloned by *in vivo* recombination in the corresponding YCpAmu1-3HA vector by replacement of the 3HA-tag. pGAL1Mep1-GFP (LEU2): the *LEU2* gene was amplified by PCR using pFL46 [74] as template and then cloned by *in vivo* recombination in the pGAL1Mep1-GFP (URA3) vector by replacement of the *URA3* gene.

**Table 3:** List of plasmids used in this study.

Plasmid	Description	Source or reference
**Low-copy-number**		
pGAL1Mep1-GFP	p416 GAL1-MEP1 (GA)_5_-GFP	This study
pGAL1Mep2-GFP	p416 GAL1-MEP2 (GA)_5_-GFP	[18]
pGAL1Mep3-GFP	p416 GAL1-MEP3 (GA)_5_-GFP	This study
YCpFL38	CEN-ARS URA3	[74]
pGAL1Mep1-GFP (LEU2)	pGAL1-MEP1 (GA)_5_-GFP-LEU2	This study
YCpAmu1	YCpFL38 AMU1	This study
YCpAmu1-3HA	YCpFL38 AMU1-3HA	This study
YCpAmu1^4R-A^-3HA	YCpFL38 AMU1^R25A,R72A,R159A,R238A^-3HA	This study
YCpAmu1^phos^-3HA	YCpFL38 AMU1^S34A,S36A,S39A,S49A,S206A,S246A,S249A,S250A,T253A^-3HA	This study
YCpAmu1-GFP	YCpFL38 AMU1-GFP	This study
YCpAmu1^4R-A^-GFP	YCpFL38 AMU1^R25A,R72A,R159A,R238A^-GFP	This study
YCpAmu1^phos^-GFP	YCpFL38 AMU1^S34A,S36A,S39A,S49A,S206A,S246A,S249A,S250A,T253A^-GFP	This study
**High-copy-number**		
YEpFL44	2μ URA3	[74]
YEpAmu1-3HA	YEpFL44 AMU1-3HA	This study
YEpAmu1^4R-A^-3HA	YEpFL44 AMU1^R25A,R72A,R159A,R238A^-3HA	This study
YEpAmu1^phos^-3HA	YEpFL44 AMU1^S34A,S36A,S39A,S49A,S206A,S246A,S249A,S250A,T253A^-3HA	This study
YEpMep1	YEpFL44 MEP1	This study
YEpMep2	YEpFL44 MEP2	This study
YEpMep3	YEpFL44 MEP3	This study

### Western immunoblotting

Total protein extracts were performed as described previously [75]. Membrane-enriched cell extracts were prepared as described previously [22]. For blot analysis, equal protein amounts (~20 μg) were loaded onto an 6% to 8% SDS-polyacrylamide gel in a Tricine system [76]. After transfer to a nitrocellulose membrane (Protran, VWR), proteins were probed with a mouse or rabbit antiserum raised against the C-terminal region of Mep1 (1:1000)[77], Mep2 (1:2000) [77], Mep3 (1:1000) [77], HA (1:10000) (Roche), GFP (1:10000) (Roche) and Pma1 (1:10000) [34]. Primary antibodies were detected with horseradish-peroxidase-conjugated anti-rabbit- or anti-mouse-IgG secondary antibodies (GE Healthcare) followed by measurement of chemoluminescence (Lumi-Light^PLUS^, Roche). For alkaline phosphatase treatment on total cell extracts, the protein pellet collected after TCA precipitation was resuspended in a solution (0.1 M de Tris-HCl pH 6.8, 20% glycérol, 4% SDS, 2% β-mercaptoethanol and 2 mM PMSF) containing proteases inhibitors (Complete Mini, Roche). This extract is then diluted 5 X in a dephosphorylation buffer [CIP 1X (Roche), 50 mM Tris-HCl 1M pH 6.8, 2 mM PMSF] containing proteases inhibitors (Complete Mini, Roche). pH could eventually be adjusted to 7.6 and the extracts were then incubated 2h at 37°C in the presence (or not) of 20 units of calf alkaline phosphatase (Roche). Proteins were precipitated with 10% TCA. For alkaline phosphatase treatment on membrane-enriched cell extracts, the collected membrane pellet was suspended in phosphatase buffer CIP 1X added of 0.1% SDS, 2 mM PMSF and proteinase inhibitors (Complete Mini, Roche). The extracts were then incubated 1h at 37°C in the presence (or not) of 10 units of calf alkaline phosphatase (Roche). Proteins were precipitated with 10% TCA. For N-glycosidase F treatment on membrane-enriched cell extracts, the collected membrane pellet was suspended in buffer (1x PBS, 10mM EDTA pH8, 0.5% octyl-glucopyranoside, 0.2% 2-mercaptoethanol, 3mM PMSF and proteinase inhibitors) and incubated 1 h at 37°C in the presence of 1.5 unit of peptide-N-glycosidase F (PNGase F, Roche).

### Co-immunoprecipitation

Co-immunoprecipitation protocol in the presence of a cross-linker was modified from [78]. About 5.10^8^ cells were washed with H_2_O, and incubated 10 min in prespheroplasting buffer (100 mM Tris pH 9.4, 40 mM β-mercaptoethanol). Spheroplasts were prepared by 30-min incubation in spheroplasting buffer (20 mM HEPES pH 7.4, 0.8 M sorbitol, 0.5X 868 medium, 40 mM β-mercaptoethanol) in the presence of 800 U of lyticase at 20°C. Spheroplasts were washed once in cross-linking buffer (20 mM HEPES pH 7.4, 0.8 M sorbitol, 100 mM potassium acetate) and resuspended in 600 μl of cross-linking buffer added with proteinase inhibitors (Complete Mini, Roche). Cross-linking was conducted with 4 mM dithiobis[succinimidylpropionate] (DSP) (ThermoScientific, 22585) for 30 min at 4°C, and the reaction was quenched by addition of 100 mM Tris pH 7.4 for 15 min at 4°C. Spheroplasts were then lysed in 200 μl of lysis buffer (50 mM Tris pH 7.4, 150 mM NaCl, 1% Nonidet P-40) added with proteinase inhibitors (Complete Mini, Roche), 2 mM phenylmethylsulfonyl fluoride (PMSF), 2 μg/ml leupeptin, 1 μg/ml pepstatin A and 0.5 μg/ml chymostatin; and broken with a glass rod and then by vortex-mixing for 5 min in the presence of glass beads. 700 μl of lysis buffer containing the different proteinase inhibitors were added and the lysates were incubated on a wheel for 30 min to extract membrane proteins. The extracts were centrifuged at 14000 rpm for 10 min. An aliquot of the lysates was collected and proteins were precipitated with 10% TCA. The remaining lysates were incubated for 30 min on a wheel with 25 μl pre-washed Pierce anti-HA magnetic beads (ThermoScientific, 88836). Beads were washed two times with 300 μl lysis buffer and proteins were eluted by incubation for 10 min at 60°C with 50 μl sample buffer (100 mM Tris pH 6.8, 4% SDS, 20% glycerol, 0.02% bromophenol blue). 50 μl of Tris 1M and 2% β-mercaptoethanol were added to the samples. Samples were analyzed by SDS-PAGE, followed by immunoblotting overnight with antibodies against HA (1:1000) (Roche) and GFP (1:1000) (Roche).

### [^14^C]-Methylammonium uptake assays

Initial rates of [^14^C]-methylammonium (BioActif) uptake were measured as described for amino acids with cells grown in minimal medium containing proline as nitrogen source. Briefly, 5-ml samples of an exponentially growing culture corresponding to about 0.25 mg protein per ml were put, without any change of the medium, into vessels containing the labelled methylammonium and preheated to 29°C or 37°C in a rotary water bath. One-millilitre samples were then removed at time intervals and poured onto filters (0.45 μM, Millipore) which were immediately washed 5 times with 2 ml iced water before counting.

### Fluorescence microscopy

Cells were observed on a Zeiss Axio Observer Z1 microscope, driven by MetaMorph (MDS Analytical Technologies). High-resolution images were captured in the confocal mode using a Yokogawa spindisk head and the HQ2 camera with a laser illuminator from Roper (405 nm 100 mW Vortran, 491 nm 50 mW Cobolt Calypso, and 561 nm 50 mW Cobolt Jive). Images were processed with Adobe Photoshop 8.0 (Adobe Systems, Mountain View, CA) and ImageJ (http://rsb.info.nih.gov/ij).
